# Towards individualised treatment of out-of-hospital cardiac arrest patients: an update on technical innovations in the prehospital chain of survival

**DOI:** 10.1007/s12471-021-01602-6

**Published:** 2021-08-09

**Authors:** J. Thannhauser, J. Nas, R. A. Waalewijn, N. van Royen, J. L. Bonnes, M. A. Brouwer, M. J. de Boer

**Affiliations:** 1 Department of Cardiology, Radboud university medical centre, Nijmegen, The Netherlands; 2 Department of Cardiology, Gelre Hospitals, Apeldoorn, The Netherlands

**Keywords:** Out-of-hospital cardiac arrest, Survival, Innovation, Technology

## Abstract

Out-of-hospital cardiac arrest (OHCA) is a major healthcare problem, with approximately 200 weekly cases in the Netherlands. Its critical, time-dependent nature makes it a unique medical situation, of which outcomes strongly rely on infrastructural factors and on-scene care by emergency medical services (EMS). Survival to hospital discharge is poor, although it has substantially improved, to roughly 25% over the last years. Recognised key factors, such as bystander resuscitation and automated external defibrillator use at the scene, have been markedly optimised with the introduction of technological innovations. In an era with ubiquitous smartphone use, the Dutch digital text message alert platform *HartslagNu* (www.hartslagnu.nl) increasingly contributes to timely care for OHCA victims. Guidelines emphasise the role of cardiac arrest recognition and early high-quality bystander resuscitation, which calls for education and improved registration at *HartslagNu*. As for EMS care, new technological developments with future potential are the selective use of mechanical chest compression devices and extracorporeal life support. As a future innovation, ‘smart’ defibrillators are under investigation, guiding resuscitative interventions based on ventricular fibrillation waveform characteristics. Taken together, optimisation of available prehospital technologies is crucial to further improve OHCA outcomes, with particular focus on more available trained volunteers in the first phase and additional research on advanced EMS care in the second phase.

## Introduction

Out-of-hospital cardiac arrest (OHCA) is a major healthcare issue, with 100–200 treated cases each week in the Netherlands [1, 2]. Survival to hospital discharge has increased to about 25%, which is relatively favourable when compared with the reported 10% worldwide [3]. Technical innovations in the prehospital infrastructure have played a key role in this process.

**Fig. 1:**
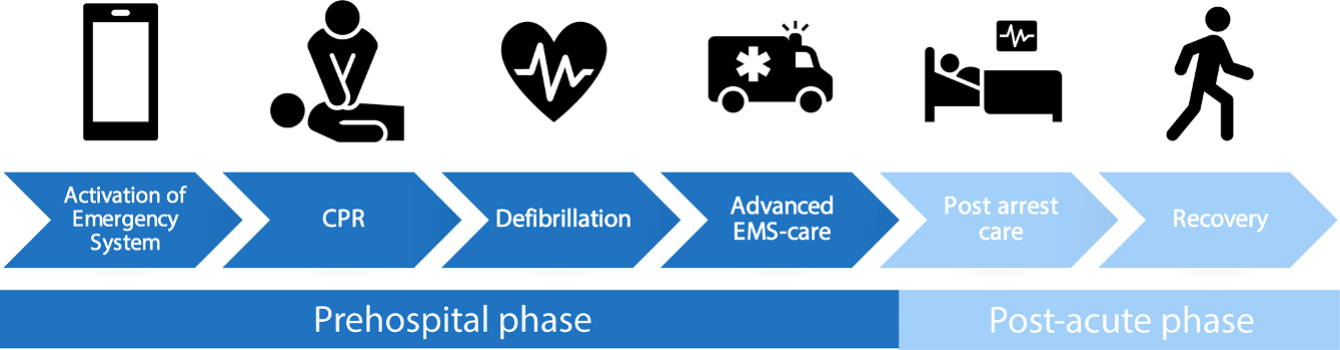
Schematic illustration of the Chain of Survival, based on the figure presented in the 2020 American Heart Association (*AHA*) guidelines for cardiopulmonary resuscitation [34]. Important links in the prehospital phase include activation of emergency medical services (*EMS*) by (lay) responders, high-quality basic life support including cardiopulmonary resuscitation (*CPR*), availability and attachment of automated external defibrillators to provide quick defibrillation, and advanced life support by healthcare professionals

The so-called ‘Chain of Survival’ defines the critical series of actions in the treatment of OHCA (Fig. 1). Crucial prehospital factors include cardiac arrest recognition by a witness, initiation of basic life support (BLS), activation of the emergency response system, access to an automated external defibrillator (AED) and advanced life support by emergency medical services (EMS).

In this article, we provide an update on technical innovations that have contributed to the optimisation of recognised prognostic factors. In addition, we highlight the most promising future techniques to further improve prehospital OHCA management.

## Bystander care

### Cardiac arrest recognition

Over the years, the impact of civilians in terms of cardiac arrest recognition and adequate initiation of BLS has been increasingly recognised. Early recognition of typical and atypical signs of cardiac arrest (e.g. gasping, uncontrolled movements) is key, in combination with early EMS activation and bystander cardiopulmonary resuscitation (CPR) until ambulance arrival.[4, 5] Educational programmes have been set up worldwide because of their potential to increase survival by higher rates of timely bystander CPR [6]. Innovative technologies such as virtual reality (VR) applications have evolved, which can present OHCA victims more realistically than standard manikins, thereby potentially improving cardiac arrest recognition. Moreover, VR training has the potential to reach a much larger target population at home and schools. Such low-cost, incomprehensible modalities have the advantage that they can be (repeatedly) used where and whenever it is convenient [7].

### Basic life support

**Fig. 2:**
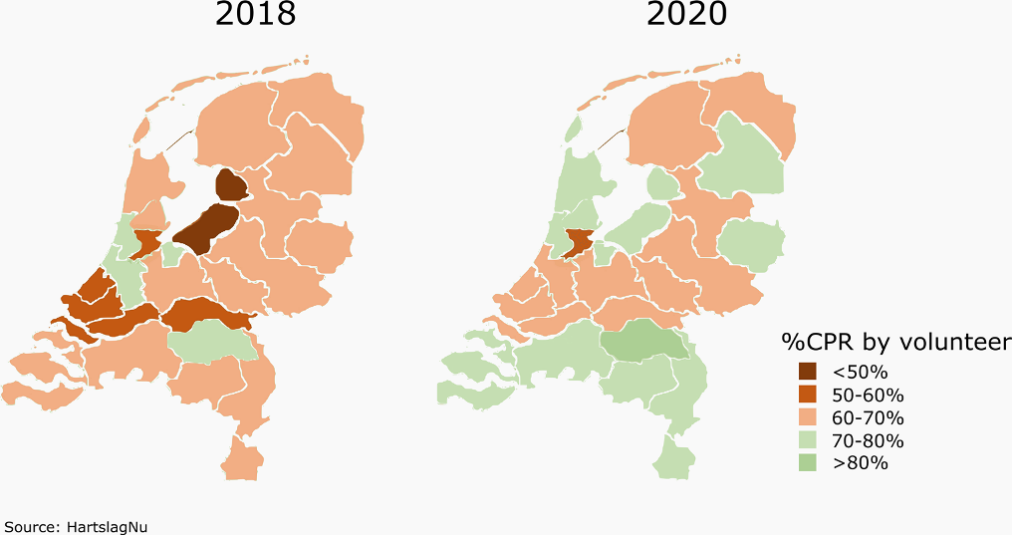
Percentage of out-of-hospital cardiac arrest cases in which initial cardiopulmonary resuscitation (*CPR*) was delivered by a trained volunteer, who arrived at the scene after receiving a message from the Dutch national text message alert system (HartslagNu). Data from all 25 Safety Regions in the Netherlands, **a** in 2018 and **b** in 2020. Source: HartslagNu. Graphic design: Contented (Apeldoorn, the Netherlands)

Besides facilitation of CPR training, technical innovations also have an important role in the optimisation of some of the key factors of survival: the rate of bystander CPR and the time to first defibrillation [8]. In an era where smartphones are ubiquitous, mobile applications have arisen to alert trained individuals to a nearby OHCA victim [8, 9]. To follow up on promising data on postal code-based applications, GPS-based systems have been developed to direct some volunteers immediately to the victim, while others are directed to the scene via the nearest registered AED [10]. To further improve the potential of this system, the Dutch Heart Foundation stimulates civilians to attend BLS training and actively register as a volunteer (*HartslagNu*, www.hartslagnu.nl), to achieve its goal that every future OHCA victim is reached within 6 min. The introduction of this digital platform has markedly facilitated CPR initiation by volunteers (Fig. 2).

To improve bystander CPR quality, guidelines now recommend witnesses to use their mobile device’s speaker function when calling the emergency telephone number [11]. This provides a unique opportunity of direct contact with dispatchers, to get real-time instructions to check for breathing, perform CPR and provide them with the precise arrest location. A future challenge appears to be developing innovative solutions to recognise unwitnessed cardiac arrest, a topic for which the Dutch Heart Foundation recently launched a project call.

### Defibrillation

Appreciating the importance of time to first defibrillation in patients with a shockable rhythm, the introduction of AEDs has significantly contributed to reduced treatment delays and improved survival.[12, 13] Over the past years, studies have been undertaken to develop and optimise shock advisory algorithms to ensure accurate, fast detection of ventricular arrhythmias [14]. Notification of the police was one of the first initiatives, which resulted in faster response times than alerting the EMS[15], but civilians may arrive even earlier with an AED [10].

Although AED availability in public buildings and areas has increased, accessibility during out-of-office hours can be improved and registration at *HartslagNu* is still suboptimal. Recently, the Dutch Heart Foundation has started a programme for AED placement in ‘optimal’ locations, with the intention of building a national AED network (www.hartstichting.nl/aed). In the city of Nijmegen, the Netherlands, an initiative of Radboud university medical centre, the municipality and volunteers has resulted in the placement of AEDs outside buildings. A novel initiative, especially in rural or remote areas, is AED delivery through drone networks [16].

## EMS care and advanced life support

### Medication

To facilitate administration of medication and/or fluids, devices have been developed for intraosseous infusion, but their effectivity is currently questioned [17]. Despite its use since the 1960s, it was not until the PARAMEDIC‑2 trial that a randomised trial demonstrated a survival benefit of epinephrine in cardiac arrest; yet, its use should be restricted to guideline-endorsed protocols [18]. Notably, ancillary analyses in this trial suggested worse neurological outcome among survivors. With available databases worldwide, big-data analyses on this topic seem warranted.

### Airway management

Over the years, a variety of innovative ventilation techniques have been developed, but their exact role needs to be further established. Insertion of supraglottic airway devices is associated with shorter chest compression interruptions than conventional endotracheal intubation, although a randomised trial did not demonstrate favourable outcomes [19]. As for laryngeal tube devices, improved 72-hour survival was reported compared with endotracheal intubation [20].

### See-through defibrillation

The EMS defibrillator has developed from a plain shock device to a ‘smart’ defibrillator. Although evidence on survival benefit is lacking, real-time feedback on CPR quality has shown to optimise chest compression rate and depth and to minimise hands-off times. A development with future potential may be ‘see-through’ defibrillation [21]. With the use of innovative filter techniques, rescuers are able to see and analyse rhythm registrations during chest compressions, aiming to further reduce hands-off times related to pauses needed for rhythm analysis.

### Ventricular fibrillation waveform analysis

The introduction of automated analysis of the defibrillator-ECG has made it possible to quantitatively characterise ventricular fibrillation (VF). While short-duration VF is known for its high frequency and high amplitude, VF of longer duration typically has low-amplitude, low-frequency characteristics. Importantly, delivery of CPR can improve VF waveform characteristics. The key VF characteristic in this context is the amplitude spectrum area (AMSA), which has been associated with myocardial energy levels and chances of successful defibrillation [22, 23].

In an ongoing randomised trial, defibrillation success is currently being compared between the prevailing resuscitation protocol and a VF waveform-guided protocol (ClinicalTrials.gov: NCT03237910). This new protocol directs prompt defibrillation in case of a high AMSA, while continued chest compressions are indicated in case of low AMSA values. In contrast to the first study on VF waveform-guided defibrillation, which showed a negative outcome,[24] this study uses a derived and validated, publicly available algorithm based on 1617 patients [23]. Notably, AMSA has also been proven to be associated with favourable neurological survival.[25] As such, it is a very early marker that may contribute to timely triage and treatment.

### Mechanical chest compressions

With the availability of mechanical chest compression devices, it has become possible to deliver long-term, sustained high-quality CPR as a safe alternative to manual chest compressions [26, 27]. Mechanical delivery of chest compressions results in higher-quality CPR in a driving vehicle, as compared with manual CPR [28]. However, its routine use does not result in superior outcomes, and mechanical chest compression delivery should therefore be tailored to specific cases and situations [27]. In particular, mechanical chest compressions may pave the way to hospital transportation for treatment of the underlying cause, e.g. by percutaneous intervention.

### Extracorporeal life support

Although the first small, randomised trial with extracorporeal membrane oxygenation (ECMO) showed promising results in patients with refractory VF [29], randomised trials such as the Dutch INCEPTION study (ClinicalTrials.gov: NCT03101787) are eagerly awaited. Infrastructure and experience are key elements, and time to initiation is crucial.[30] With ongoing technical developments, some research groups are now able to provide prehospital treatment with portable extracorporeal CPR devices. Further identification of groups that may derive particular benefit is warranted [30, 31].

## Future directions: Optimise what we have, develop what we need

The development of new resuscitative options raises the question whether OHCA treatment should become more patient-tailored. In general, all OHCA patients are treated the same, regardless of the clinical scenario or underlying cause.

The conventional ‘stay and play’ strategy—on-scene CPR until return of spontaneous circulation is achieved—may need refinement, with conversion to a ‘scoop and run’ strategy in well-defined situations. Especially in case of VF refractory to standard in-field treatment, early transportation with mechanical chest compressions and timely ECMO may be of value. The first initiatives of routine early transport to the catheterisation lab in case of refractory VF reported high rates of underlying coronary substrate and promising clinical outcomes [29]. The recent COACT trial showed that routine coronary angiography does not improve survival in VF patients with return of spontaneous circulation and no signs of ST-elevation myocardial infarction (STEMI) [32]. Hence, the actual challenge seems to be identifying patients with an acute coronary occlusion in the prehospital setting.

At present, detection of STEMI is restricted to those who regain organised rhythm, which is a subgroup of patients who are often in a later phase of the arrest. One of the potential new technologies that may result in early identification of patients with a myocardial infarction is VF waveform analysis. In experimental work, the first in-human study has suggested that this technique may not only predict defibrillation success and survival but also underlying infarction [33]. Future clinical studies on this topic are warranted.

## Conclusion

Technical innovations have played a role in the positive survival trend for OHCA in the Netherlands, with current survival rates of roughly 25%. With the growing accessibility of smartphones, performance of bystander CPR and availability of AEDs at the scene have substantially improved. Registration of AEDs and trained volunteers registered on national text message alert platforms are crucial to reach the full potential of this lifesaving, mobile network. For EMS care, developments such as mechanical chest compression devices and ECMO and the future potential of ‘smart’ defibrillators may pave the way towards more individualised treatment approaches for cardiac arrest.
